# Arc-induced Long Period Gratings in standard and speciality optical fibers under mixed neutron-gamma irradiation

**DOI:** 10.1038/s41598-017-16225-4

**Published:** 2017-11-20

**Authors:** Andrei Stancălie, Flavio Esposito, Rajeev Ranjan, Petrişor Bleotu, Stefania Campopiano, Agostino Iadicicco, Dan Sporea

**Affiliations:** 1 National Institute for Laser, Plasma and Radiation Physics, Center for Advanced Laser Technologies, 409 Atomiştilor St., Măgurele, RO-077125 Romania; 2 Department of Engineering, University of Naples “Parthenope”, Centro Direzionale Isola C4, 80143 Naples, Italy; 3 “Politehnica” University, Bucharest, Splaiul Independenţei 303 060042 Romania

## Abstract

In this paper, for the first time, the effects of mixed neutron and gamma flux on the spectral and sensing responses of Long Period Gratings (LPGs) are thoroughly analyzed. Six LPGs written by means of Electric Arc Discharge (EAD) technique in standard and speciality fibers, including radiation-hardened ones, were tested. The EAD technique was chosen because it enables the writing of gratings both in standard and not photosensitive fibers. The experiments have been carried out in a “TRIGA” pulsed nuclear reactor and the LPGs were irradiated by a gamma-ray dose-rate of 9 Gy/s and a mean 1.2∙10^12^ n/(cm^2^s) neutron flux. Real time monitoring was performed for a comparative investigation of LPGs’ response, in terms of radiation sensitivity and wavelength shift. Experiments show that LPG in a radiation-resistant fiber exhibits resonant wavelength shift higher than LPG in standard fiber. The changes of temperature sensitivity due to radiation were experimentally established by comparison of pre- and post-radiation characterization, indicating that radiation effects induce a slight increase of the temperature sensitivity, except for the LPG in pure-silica fiber. Theoretical and numerical analysis was combined with experimental data for evaluation LPGs’ parameters changes, such as refractive index and thermo-optic coefficient, after exposure to radiation.

## Introduction

Over the years, great efforts have been made in order to enhance the sensitivity of in-fiber sensors to external parameters like temperature, strain, humidity, pressure and so on, with the aim to enlarge their field of application. More recently, their use in radioactive environments attracted a growing interest for research, industrial and medical purposes^1,2^. In fact, optical fiber sensors operating in ionizing radiation fields have been studied in several research papers, with respect to the radiation effects on their properties^2–4^ and for radiation dosimetry^5^. A critical aspect is the assessment of their resistance under different radiation conditions, ranging from low radiation levels, typically present in medical applications, to high-energy applications. For these reasons, the research focused on the exposure of optical fibers and fiber sensors to gamma-ray^6–8^, charged particles^9,10^, synchrotron^11^ and X-ray^12^ radiation. The most part of the studies targeted Fiber Bragg Gratings (FBGs), fabricated in different optical fibers (photosensitive, H-loaded, or rad-hard) and investigated both in gamma and neutron-gamma mixed environment^2,4,13–15^. Concerning Long Period Gratings (LPGs), there are only few investigations reporting their behaviour under gamma irradiation. One of the first experiments was conducted by Vasiliev *et al*.^16^ in 1998 using LPGs inscribed by CO_2_ laser in N-doped optical fiber, and by UV radiation in Ge-doped optical fiber. More recently, Rego *et al*. presented a first evidence of LPGs by electric arc discharge in rad-hard fiber, when irradiated by a gamma source^17^. Some other works, dealing with specially designed LPGs, reported their high sensitivity to gamma radiation: chiral type LPGs from five different suppliers by Henschel *et* *al*.^18^, and turning point LPG written in B/Ge co-doped fiber by means of CO_2_ technique by Kher *et* *al*.^19^. In all the cases, the gamma irradiation induced changes in the refractive index of the optical fiber (with typical values around 10^−3^) as consequence of the generation of defects, and affecting mostly the doped regions of the optical fibers. Such changes resulted in wavelength shifts of the LPGs’ attenuation bands that, despite their magnitudes were dependent on the irradiation dose and type of fiber, did not show any particular behaviour associated to their particular fabrication technique.

It is important to remark here that there exists a difference between the dependence of the radiation effects on FBGs and LPGs. Indeed, the wavelength shift recorded by FBGs is related to the induced changes in the core effective refractive index, whereas for LPGs it is related to the difference between core and cladding effective refractive indices. Moreover, even if in the case of FBGs it was observed that the radiation effects are usually dependent on fiber composition, dose-rate, and temperature^1,14^. Until now there is no systematic investigation on LPGs behaviour under irradiation, mostly due to the high irradiation costs.

On this line of argument, the authors were focused on the experimental and numerical investigation of the gamma radiation induced effects on LPGs fabricated in different fibers and by means of various techniques, studying both their resonant wavelength shift as function of radiation dose and their temperature sensitivity changes, as both are two known effects caused by irradiation^2^. In ref.^20^, a LPG manufactured in pure-silica core with F-doped cladding fiber, being produced by CO_2_ method, was studied when irradiated with a dose rate of 0.2 kGy/h up to a total dose of 45 kGy. A low radiation sensitivity was found, with induced shifts of the resonance wavelength lower than 1 nm. The radiation induced shift recovery was almost complete in about 120 h, at room temperature. A comparative report was presented in ref.^21^, focused on LPGs written by CO_2_, FBGs and draw tower gratings (DTGs) irradiated under the same conditions. More recently, we focused on the effects of gamma radiation on LPGs written by Electric Arc Discharge (EAD)^22^, by a comparative analysis of two LPGs in standard fibers and one in radiation resistant fibers (from Nufern). The gratings were irradiated under gamma with a dose rate of 0.18 kGy/h with a final dose of 35 kGy. It was found that resonant wavelength shifts are strongly dependent on the hosting fiber, obtaining a maximum sensitivity of 0.73 nm/kGy for LPGs in standard fiber, and up to 1.34 nm/kGy for Nufern fiber. Concerning the temperature sensitivity, we measured a slight decrease due to radiation exposure. Moreover, a novel approach was developed where experimental results were combined with numerical simulations, in order to quantify the radiation-induced effects on optical fibers, in terms of refractive index and thermo-optic parameter change. A maximum increase of the core refractive index of 2.5∙10^−5^ concerning the Nufern fiber, and a maximum change of the thermo-optic coefficient of 1.5∙10^−8^/°C concerning the standard fiber were estimated.

However, since the interest of the scientific community in the in-fiber devices for radiation environments is huge, despite the mentioned results, further work is still required to have a complete knowledge and understanding of optical fibers sensors behaviour in nuclear environment. In this sense, LPGs are an important subject to investigate, and, to the best of our knowledge, these sensing devices were never tested before under neutron irradiation. For these reasons, in the present paper we propose a wide experimental and numerical comparative investigation, concerning the effects on six LPGs written by EAD technique in different fibers, when exposed to a mixed gamma-neutron radiation field, at the nuclear reactor operated by the “Nuclear Research Institute ICN” (Mioveni, Romania). In particular, the attention has been focused on: (i) two standard Ge-doped SMF28 fibers provided by OZ Optics and Thorlabs, (ii) a speciality fiber for harsh environments (whose manufacturer and model are not reported here for confidentiality reason), (iii) the radiation resistant R1310-HTA manufactured by Nufern, and (iv) the pure-silica core with F-doped cladding DrakaSRH fiber from Prysmian-Draka. The LPGs were subjected to a gamma dose rate of 9 Gy/s and a mean 1.25∙10^12^ n/(cm^2^s) neutron flux for about two hours, resulting in a total dose of 64.8 kGy and a neutron fluence of 9.18∙10^15^ n/cm^2^. The wavelength shift of the LPGs was monitored in real-time during the exposure. Moreover, the changes in optical fiber refractive index and thermo-optic coefficient were also calculated by combining the experimental results with numerical simulations. As a result, a comparison between the gamma-neutron induced changes in gratings fabricated in fibers with different composition is provided.

### Fabrication of Long Period Gratings

In order to investigate the effects of mixed neutron-gamma radiation on LPGs, and their parameters dependence on the fiber hosting the grating, several LPGs were fabricated in different optical fibers. In particular, we selected standard Ge-doped fibers (Corning SMF28 type) and speciality optical fibers, declared by their manufacturers as designed for harsh environments or to be radiation resistant.

The Electric Arc Discharge technique is a good candidate for the fabrication of LPGs both in standard and non-conventional fibers^17,23,24^. Recently, we have successfully applied this technique to several speciality fibers, such as pure-silica core^25^, Phosphorus-doped^26^, hollow core^27,28^, and polarization-maintaining^29,30^ fiber. A detailed report of the fabrication setup can be found in refs^24,25^. Briefly, a periodic perturbation is achieved through the application of arc discharges to the fiber, provided by Sumitomo Type-39 fusion splicer, with typical arc parameters: power in range of 1–15 step (proprietary unit) and time of 200–900 ms. In particular, for the aim of current paper, the power was selected in range 1–5 step and the time was 380–460 ms, resulting in devices with final length shorter than 25 mm. During the procedure, one end of the fiber was fixed to a precision translation stage (with resolution better than 1 μm), while the other end was kept under constant axial tension, which was obtained in this case by using a 12 g weight. The desired spectral features of the LPG are obtained after the application of a sequence of arc discharges to the uncoated fiber, with the discharges being alternated with fiber displacement by the grating period. The period of the gratings was changed depending on the hosting fiber, to tune the position of the attenuation band related to the LP_04_ cladding mode around 1560 nm. The stability and repeatability of the fabrication platform was such that the difference between the wavelength positions of samples, realized using the same parameters, is in range ±4 nm.

**Figure 1:**
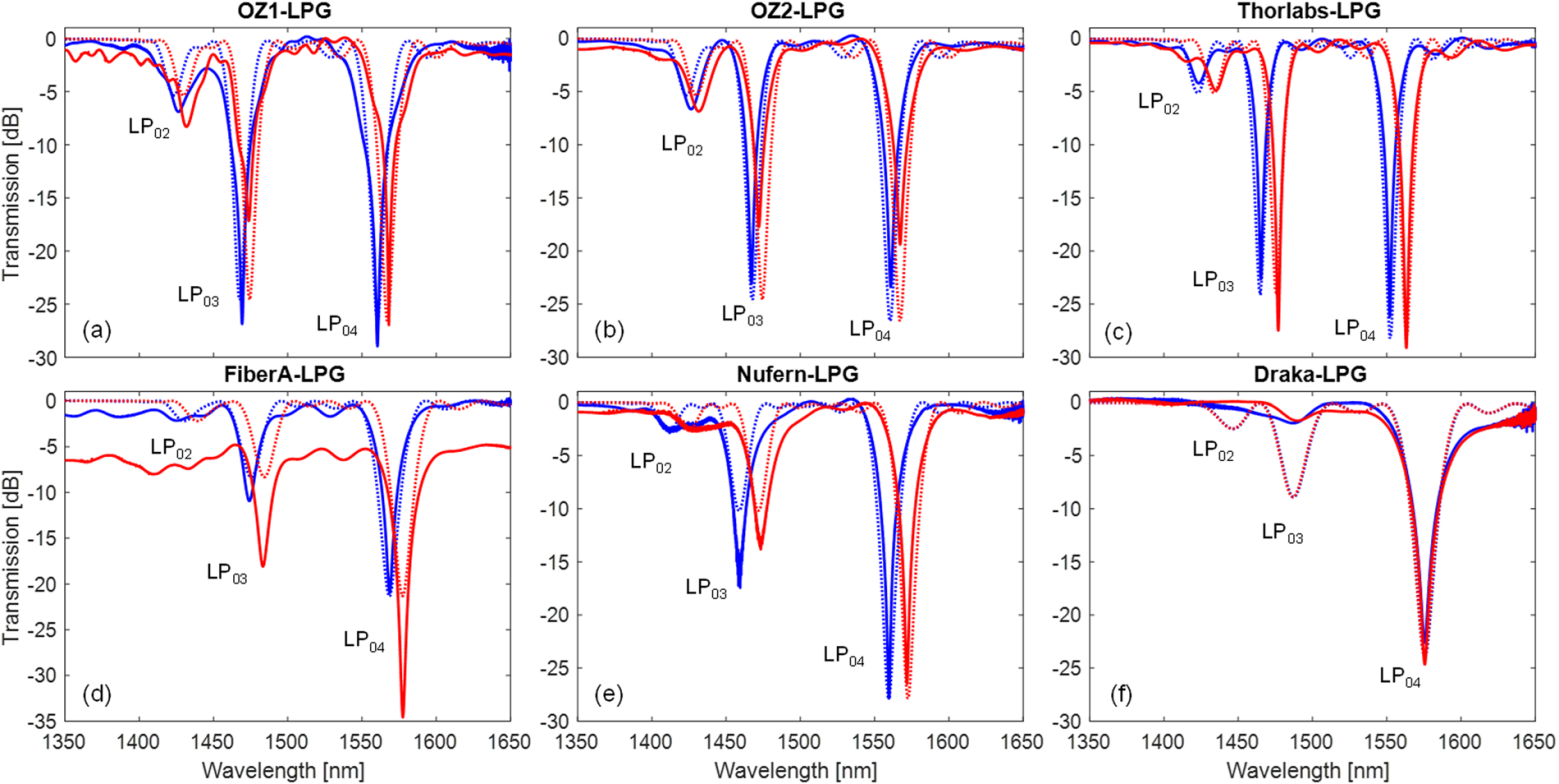
Transmission spectra before irradiation (blue) and after irradiation (red), and comparison between experimental (solid) and numerical (dotted) data for: (**a**) OZ1-LPG; (**b**) OZ2-LPG; (**c**) Thorlabs-LPG; (**d**) FiberA-LPG; (**e**) Nufern-LPG; (**f**) Draka-LPG.

Off line broadband gratings’ transmission spectra are plotted in Fig. 1 as blue solid lines and were recorded with OSA Yokogawa AQ6370B (resolution set to 0.1 nm), the illumination being provided by a broadband source (involving several SLEDs in the range 1100–1700 nm). Resonant wavelengths were computed via centroid analysis proving a resolution of ±5 pm. In the same figure, the experimental data are compared with numerical spectra (reported as blue dotted lines), obtained by coupled-mode theory (CMT)^31,32^, resulting in a good agreement. This accurate model allows the calculation of the LPG’s spectrum, given the properties of the hosting fiber and grating parameters: (i) fiber geometry, (ii) core and cladding dispersive refractive index, (iii) external medium refractive index, (iv) grating modulation strength, period, and length.

The details of the optical fibers and LPGs are the following:

I. Ge-doped SMF28 fibers having D_core_ = 8.2 µm, D_clad_ = 125 µm, MFD = 10.4 ± 0.8 µm @ 1.55 µm, and NA = 0.14 supplied by OZ Optics and Thorlabs, respectively. For the purpose of this investigation, we fabricated two LPGs with the same period of Λ = 630 μm in OZ fiber, resulting in LP_04_ centered at 1560.4 nm and 1560.8 nm, with depth of 29.0 dB and 23.4 dB, respectively, and a LPG with Λ = 646 μm in Thorlabs fiber having LP_04_ band at 1552.1 nm with depth of 26 dB. We have to underline that, even if both manufactures (OZ Optics and Thorlabs) claim that their products are in compliance with Corning SMF28, the fibers exhibit some physical and/or structural differences (probably due to slightly different dopant concentration, residual stress state, or due to the tolerance of drawing process). This fact imposes a slight tuning in the grating period. The gratings’ spectra are plotted in Fig. 1a,b and c being identified as OZ1-LPG, OZ2-LPG, and Thorlabs-LPG, respectively.
II. FiberA, having D_clad_ = 125 µm, MFD = 10.4 ± 0.8 μm @ 1.55 µm, and NA = 0.12, is declared by the manufacturer to be suitable for harsh environments. Here the manufacturer and the fiber model are not reported for confidentiality reason. Fiber A-LPG was fabricated with a period Λ = 625 μm, having LP_04_ centered at 1568.6 nm with depth of 21.0 dB, see Fig. 1(d).
III. R1310-HTA fiber manufactured by Nufern, with D_core_ = 9.0 µm, D_clad_ = 125 µm, MFD = 10.5 ± 1.0 μm @ 1.55 µm, and NA = 0.12. This fiber is declared by the manufacturer as optically and mechanically similar to SMF28 but with improved radiation performances^33^. Nufern-LPG was fabricated in this fiber with a period Λ = 677 μm, resulting in LP_04_ centered at 1559.6 nm with depth of 27.8 dB, as shown in Fig. 1(e).
IV. Pure-silica core with Fluorine-doped cladding DrakaSRH fiber manufactured by Prysmian-Draka, having D_core_ = 9.0 µm, D_clad_ = 125 µm, and MFD = 10.1 ± 0.5 μm @ 1.55 µm. This fiber is optimized for use in highly radiative environments, due to its pure-silica core. The spectrum of Draka-LPG, fabricated with a period Λ = 540 μm, and having an attenuation band centered at 1575.5 nm with depth of 22.6 dB, is shown in Fig. 1(f). From numerical simulations, it is reasonable to believe that this attenuation band is associated to LP_04_ as well, despite the fact that for this fiber the agreement between experimental and numerical data is very good for the band under analysis and less for the others. In particular, on the experimental curve, just one attenuation band is visible for lower wavelengths. This could be attributed to high power losses in the cladding modes (which cannot be taken into account in simulation) leading to a lower depth of the LP_03_ mode and a complete suppression of the LP_02_ mode.

Finally, we would also like to mention that, for each fiber, several samples were prepared in order to identify the fabrication parameters leading to stable and repeatable results. However, due to high irradiation costs, we had a “limitation” in the number of samples actually irradiated. For these reasons, we decided to consider two similar samples in SMF28 fiber (for repeatability) and the other samples all fabricated in different fibers, in order to investigate as much as possible the relationship between the results and fiber type.

### Experimental setup and methods

The irradiation was performed at the research nuclear reactor from the “Nuclear Research Institute ICN”, Mioveni, Romania. The optical fibers were placed inside the central channel of the “TRIGA ACPR” reactor, operated in stationary mode, and whose power was stabilized to 100 kW during the irradiation.

**Figure 2:**
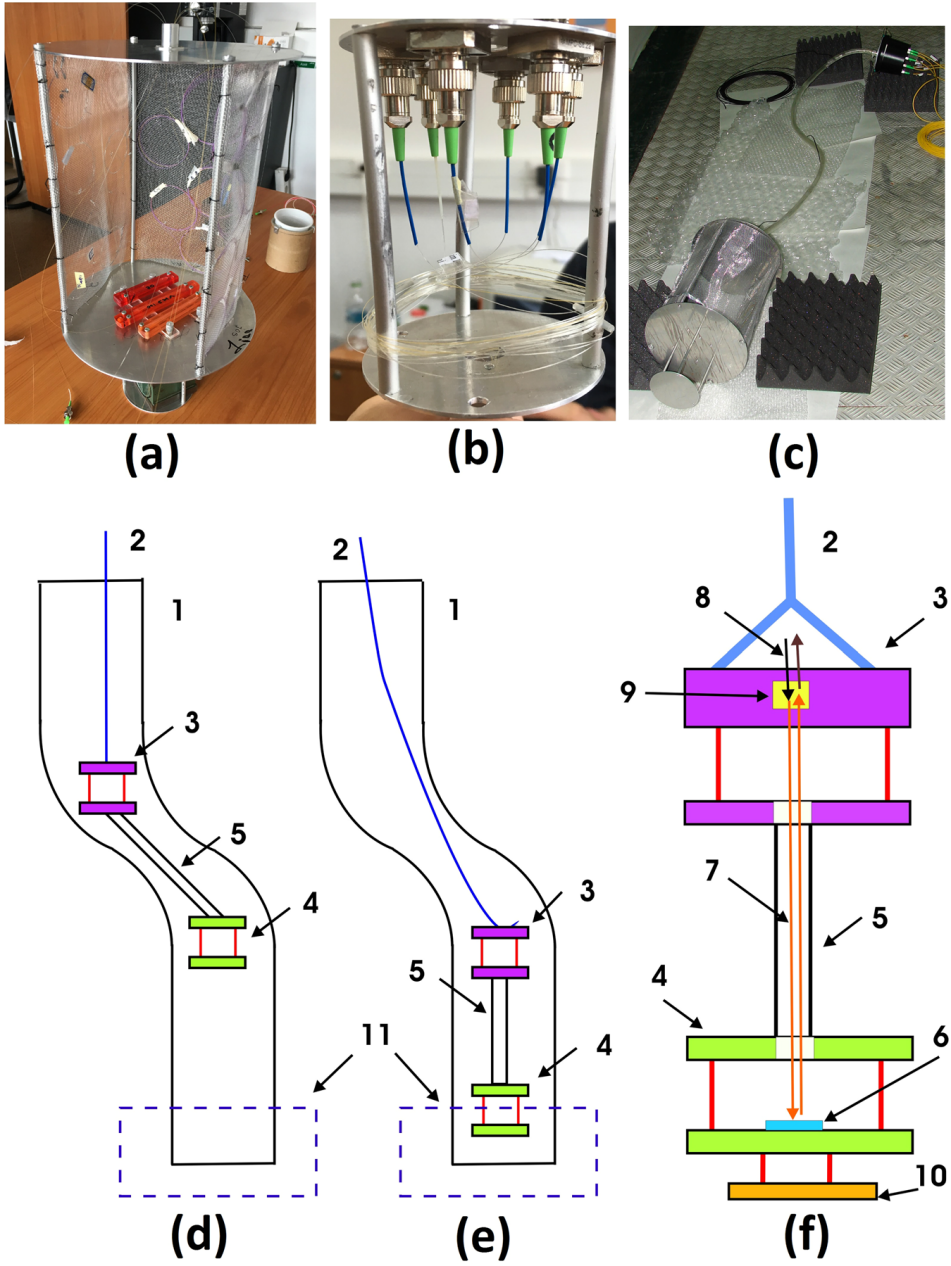
Photos of the experimental setup holder for fibers: (**a**) lower cage housing the frames with LPGs; (**b**) upper cage with connectors; (**c**) the complete assembly. The sketch of the setup and the irradiation chamber (not at scale): (**d**) the position of the carrying cages during the descending operation; (**e**) the position of the cages during radiation exposure; (**f**) the simplified design of the two cages; 1 – irradiation channel; 2 – textile rope; 3 – upper Al cage; 4 – lower Al cage; 5 – flexible plastic tube; 6 – one of the LPGs samples; 7 – LPG’s optical fiber; 8 – connecting optical fibers for on-line measurements; 9 – fiber adaptors for connecting optical fibers; 10 – spacer for cage positioning on the irradiation channel bottom; 11 – irradiation zone.

Before the irradiation, a bespoke setup was developed with the aim to properly arrange both optical fibers and LPGs during their deployment in the irradiation zone, since the operator has no direct access to it for safety reasons. Each grating is arranged in a special frame in order to ensure constant strain state during the experiment. Frames containing the sensors are fixed on the bottom part of a cylinder cage made of aluminum alloy, as in Fig. 2(a). The cage has two discs connected together with aluminum bars. Optical fibers go through a small space crafted in the center of the upper disc. The 3 m long fibers (of both ends of each grating) with connectors reach the support of a second cage hosting the adapters, as in Fig. 2(b), to couple the sensors to 20 m long fiber patch cords, necessary to reach the sensors interrogation unit. The two parts, the lower one with the sensors and the upper one with the adaptors, are separated by 2 m long fibers protected by a plastic flexible tube (see Fig. 2(c)), in order to avoid the activation of the connectors and adaptors during the irradiation. The mechanical parts were built with aluminum as this has a shorter activation decay time.

In order to be placed in the proximity of the radiation source, the setup has to be deployed down in a tube with a diameter of 35 cm, located eight meters underground, in a water tank. As reported in Fig. 2(d), the tube has an S-shape form in front of the irradiation zone (which is 40 cm in length) to limit the radiation escape outside. The setup was designed with respect to the *in-situ* geometry so that the two cages containing the sensors and the optical fiber adapters may pass through the offset and the lower cage be positioned in the irradiation zone, whereas the metal adaptors fixed in the upper cage are kept out of the irradiation zone and shielded (Fig. 2(d–f)). The sketches in Fig. 2(d–f) are not represented at the real scale.

The sensors were irradiated at the nuclear reactor during two different sessions, by keeping the same irradiating conditions. Two identical setups were employed, each one including three LPGs. The sensors were deployed inside the reactors’ central channel with the reactor off-line, after that both measuring instrumentation and reactor are turned on and shut down after a two hours irradiation session. Real-time monitoring was performed by using a 4-channel SM125 optical interrogator from Micron Optics. Each grating is connected to a channel of the instrument through an optical circulator and appropriate patchcords. The interrogator allows automatic recording of the central wavelengths’ changes with 1 Hz maximum frequency. For safety reasons, the equipment was placed outside the irradiation area in a radiation free zone located 20 m away.

Dose measurements were performed for both gamma and neutron irradiation with standard methods. For gamma radiation estimation, MCNPX 2.6.0 was utilized, the model requiring a 20 ml volume of liquid absorbent placed near the sensors. The method indicated a 9 Gy/s dose-rate for 100 kW reactor power. The residual gamma dose was calculated by “LABORAD” laboratory using spectrophotometry UV-VIS method and a Frick dose-meter resulting in a 8.5 Gy/h dose rate. For neutron flux, spectra and axial distribution, the method of gold monitor activation was applied, that implies knowing the neutron spectra and the mean interaction section value. To estimate the absolute flux and the neutron spectra from the measured reaction rates, the unfolding technique was applied by using the SAND-II code. The results indicated a neutron flux of 1.255∙10^12^ n/(cm^2^s) for 2 hours irradiation time, giving a neutron fluence of 9.18∙10^15^ n/cm^2^ at a reaction rate of 6.526∙10^−11^. In the following section, the experimental and numerical simulation results are presented in a comparative manner.

## Results

### Spectral characterization and analysis

In this section, we report on the radiation induced effects on LPGs, focusing the attention on the measured variation in their spectrum. Concerning the irradiation sessions: OZ1-LPG, Thorlabs-LPG, and Nufern-LPG were irradiated during the first session, whereas OZ2-LPG, FiberA-LPG, and Draka-LPG during the second one. For both sessions a dose rate of 9 Gy/s, a total gamma dose of 64.8 kGy was reached after two hour experimentation, whereas the mean neutron flux was 1.25∙10^12^ n/(cm^2^s) resulting in a neutron fluence of 9.18∙10^15^ n/cm^2^.

The experimental transmission spectra of the irradiated LPGs are reported in Fig. 1 as red solid lines, in comparison with original spectra before irradiation. As one can observe the most significant effect is related to the change in the position of the resonance wavelengths; in fact all the attenuation bands exhibited a red shift that is significantly dependent on the fiber hosting the grating, except for Draka-LPG. In particular, concerning the LP_04_ band, similar wavelength shifts of 6.37 nm and 6.40 nm were measured for OZ1-LPG and OZ2-LPG, respectively. The agreement found in the wavelength shift of the two OZ gratings, which were irradiated in different sessions, is a proof of the repeatability of the irradiation conditions. Although it is reported as a Corning SMF28 fiber, Thorlabs-LPG exhibits a surprising wavelength shift of 10.91 nm during the same irradiation session. Concerning the grating written in the special fiber for harsh environmental applications and radiation resistant fiber (FiberA-LPG) and Nufern-LPG, shifts of 8.99 nm and 11.75 nm were measured, respectively. Differently, for the grating written in pure silica core fiber, Draka-LPG, a trivial blue shift of 0.39 nm was noticed, as expected from the hosting fiber composition.

According to considerations reported in previous papers^2,13,22,34,35^ concerning the effects of gamma and neutron-gamma irradiation on optical fiber and devices, it is expected that the radiation generates/modifies defects in the structure of the optical fiber, resulting in an increase of its refractive index. Thus, the refractive index of core and cladding after the irradiation can be schematically expressed as:

1 $$\{\begin{array}{c}{n}_{co}(\lambda )={n}_{co,0}(\lambda )+{\rm{\Delta }}{n}_{co}^{irr}\\ {n}_{cl}(\lambda )={n}_{cl,0}(\lambda )+{\rm{\Delta }}{n}_{cl}^{irr}\end{array}$$

where *n*_*co*,0_ and *n*_*cl*,0_ represent the core and cladding refractive index of the pristine fiber, respectively, whereas ${\rm{\Delta }}{n}_{co}^{irr}$ and ${\rm{\Delta }}{n}_{cl}^{irr}$ represent the radiation induced changes of core and cladding refractive index, respectively. Such changes are promoted by the presence of dopants in the optical fiber, with different effects for different kind of doping even for sub-mol% levels. Thus, we considered as trivial the refractive index changes in the fiber region with pure-silica composition, which can be either core or cladding depending on the optical fiber.

In this case, the changes in refractive index cannot be measured by means of direct techniques, as refracted near-field, quantitative phase microscopy, differential interference-contrast microscopy, computerized tomography, multiwavelength interferometry, etc.^36^. In fact, additional challenges have to be considered: (i) the RI should be measured in the fiber at the location of the LPG, as it can be changed both by the writing process and the irradiation; (ii) the measurement requires the cut of the fiber (i.e. the analysis is destructive); (iii) as post irradiation recovery can occur, the real changes of the RI have to be linked with real-time measurements.

Based on these considerations and bearing in mind the wavelength shifts recorded at the end of irradiation, a reverse-engineering procedure was applied in order to estimate the corresponding refractive index change, where the different parameters of each LPG and fiber were considered. In particular, changes were calculated to be ${\rm{\Delta }}{n}_{co}^{irr}$ = 2.6∙10^−5^ for OZ fiber, ${\rm{\Delta }}{n}_{co}^{irr}$ = 4.1∙10^−5^ for Thorlabs, $\Delta {n}_{co}^{irr}$ = 3.5∙10^−5^ for FiberA, and ${\rm{\Delta }}{n}_{co}^{irr}$ = 4.1∙10^−5^ for Nufern, whereas for the same fibers it was considered ${\rm{\Delta }}{n}_{cl}^{irr}$ = 0. Concerning Draka fiber, a change in cladding refractive index ${\rm{\Delta }}{n}_{cl}^{irr}$ = 0.2∙10^−5^ was extrapolated, since the core region is made of pure-silica and thus $\Delta {n}_{co}^{irr}$ = 0. Accordingly, the numerical spectra of post-irradiated gratings obtained using these parameters were also plotted in Fig. 1 as red dotted lines, showing a good agreement between experimental and numerical data. The results on wavelength shifts at the end of irradiation and refractive index changes for all the fibers are summarized in Table 1.

**Table 1:** Summary of radiation induced effects on LPGs produced in different fibers.

Sensor id	Optical Fiber		Experimental wavelength shift	Numerical	
	Model	Type		Core RI change ${\rm{\Delta }}{n}_{co}^{irr}$	Cladding RI change ${\rm{\Delta }}{n}_{cl}^{irr}$
OZ1-LPG	SMF28	Standard	6.37 nm	2.6∙10^−5^	—
OZ2-LPG	SMF28	Standard	6.40 nm	2.6∙10^−5^	—
Thorlabs-LPG	SMF28	Standard	10.91 nm	4.2∙10^−5^	—
FiberA-LPG	N.A. (confidential)	Specialty fiber (for harsh environment)	8.99 nm	3.5∙10^−5^	—
Nufern-LPG	Nufern R1310-HTA	Specialty fiber (radiation tolerant)	11.75 nm	4.1∙10^−5^	—
Draka-LPG	DrakaSRH	Pure-silica core, F-doped cladding	−0.39 nm	—	0.2∙10^−5^

### On-line results

In the previous section, we considered the only shifts in LPGs’ resonance wavelengths at the end of the irradiation. In addition, a deeper study can be carried out by the real-time monitoring of the wavelength shift as function of the radiation exposure time.

**Figure 3:**
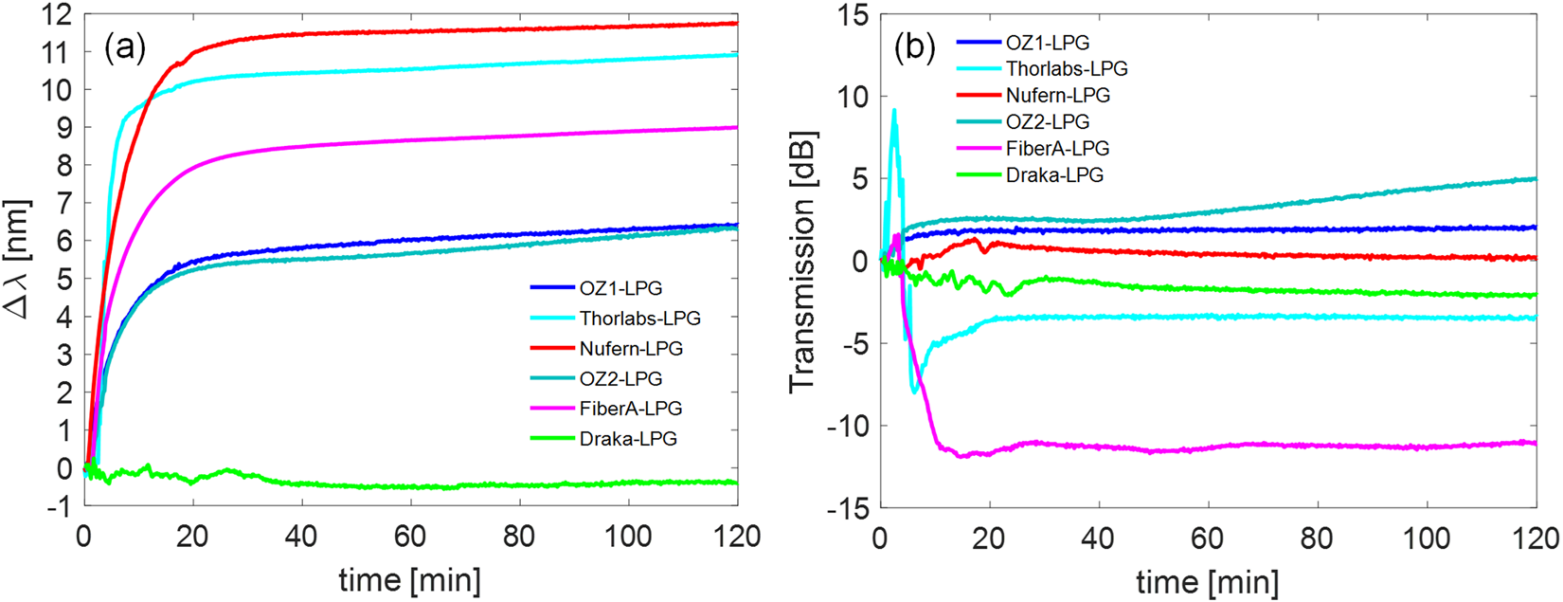
Measured variation of the (**a**) spectral position and (**b**) transmission of the LP_04_ resonance wavelength with time during the irradiation.

Figure 3(a) plots the shifts of the LP_04_ resonant wavelengths of the LPGs, whereas in Fig. 3(b) the change in transmission is reported for the same band. As one can observe from Fig. 3(a), wavelength shift monotonically increases with time, i.e. with gamma dose and neutron fluence, experiencing higher sensitivities during the first minutes of irradiation. Moreover, saturation behaviour can be observed for all gratings after about 30 minutes, where the shift was already higher than 90% of the final value, and corresponding to a gamma dose of 16.3 kGy and 2.295∙10^15^ n/cm^2^ neutron fluence.

In Fig. 3(a), it is important to observe that both OZ gratings exhibit a very close wavelength shift behaviour for the entire radiation experiment. Differently, Thorlabs-LPG exhibits significantly higher wavelength shift than OZ gratings. Thorlabs-LPG also shows maximum shift rate versus time in the first few minute (in particular in range 2–6 minutes). It is reasonable to believe that the different radiation behaviour of OZ and Thorlabs fibers is due to tolerance of the fabrication process. Moreover, since similar radiation induced wavelength shifts were recorded for both fibers when exposed to only gamma^22^, the difference in this case could be principally attributed to the presence of neutrons.

Besides, the results of OZ-LPGs and Nufern-LPG are in agreement with trends of our previous results^22^, where similar LPGs in the same fibers were irradiated under gamma up to 35 kGy, at the dose rate of 0.05 Gy/s. In particular, the wavelength shifts recorded in Nufern fiber is about 1.8 times higher than in standard OZ fiber, similarly to what is reported in previous work^22^. This is possible by assuming that variation of the difference between effective refractive indices of core and cladding modes in Nufern fiber is higher than in the standard fiber, and thus the radiation generates different defects in this fiber due to the doping level (indeed the two fibers have different NA). It should be remarked that, concerning the fact that Nufern R1310-HTA fiber is declared as “radiation resistant”, such feature is primarily related to its RIA (radiation induced attenuation) as demonstrated in ref.^33^. Anyway, it should be mentioned that often no direct correlation was found between the induced absorption and the refractive index change^2,18,37^. Concerning FiberA, the different value of NA with respect to the standard fiber would suggest also in this case a different doping concentration. Unfortunately, the manufacturers are usually not keen in providing exact information on the chemical composition of their fibers and the manufacturing technologies used, which make, in some cases, difficult a correlation of radiation effects to the optical fiber type. Finally, the trivial wavelength shift recorded by Draka-LPG confirmed the suitability of such fiber for hard radiation environments with neutron as well, since it has pure-silica core^38^.

Concerning the power transmission of the LP_04_ band, as reported in Fig. 3(b), changes were noticed in the range ±5 dB. The only exception is FiberA where a change in the power of the baseline was also noticed, as it can be seen from Fig. 1(d). The changes in transmission can be related to changes in cladding modes coupling coefficient, as a consequence of the radiation induced refractive index changes.

### Temperature Sensitivity changes

A comparison between the temperature response of the LPGs before and after the irradiation was also done, since it was observed that radiation affects this parameter as well^20–22^. The characterization was performed by using a setup similar to the one described in ref.^25^, the temperature monitoring was done using a commercial FBG-based sensor and the temperature range investigated was 25–150 °C.

**Figure 4:**
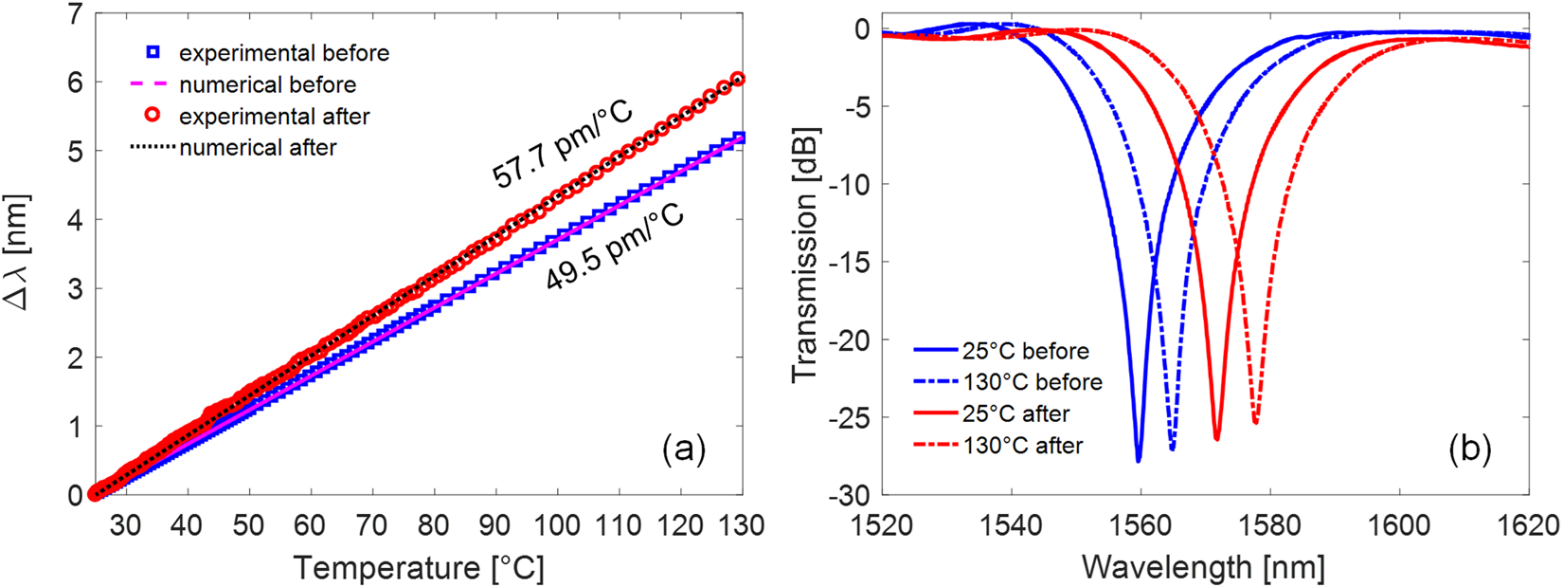
LP_04_ band of Nufern-LPG: (**a**) measured spectral position variation with temperature, before and after irradiation, as compared with simulations. (**b**) Spectra at room temperature and at high temperature, before and after irradiation.

First, we would like to highlight that, due to the radiation; all the gratings exhibited a change in their thermal sensitivities. As example, in Fig. 4(a) the temperature behaviour of the LP_04_ band of Nufern-LPG is reported before and after irradiation (with markers), as compared with numerical results (lines). Whereas in Fig. 4(b) it is reported the comparison between the spectra related to the same band at room temperature and at higher temperature (130 °C), both for measurements done before and after irradiation. Similar results were found for the other gratings. In particular, concerning OZ1-LPG, Thorlabs-LPG, and FiberA-LPG the sensitivity of LP_04_ increased of about 2%, passing from the value of 50.0, 47.6, and 50.8 pm/°C before the irradiation to 51.2, 48.6, and 51.6 pm/°C, respectively, after irradiation. Concerning OZ2-LPG, the sensitivity before irradiation was similar to OZ1-LPG (49.9 pm/°C), whereas it was not possible to perform post-irradiation characterization since the grating was accidentally broken during the temperature setup preparation. Concerning Nufern-LPG, a greater increase in the thermal sensitivity was recorded, changing from 49.5 to 57.7 pm/°C (plus 17%) for LP_04_. Whereas for Draka-LPG a 10% decrease in the sensitivity was recorded, passing from the value of 29.6 to 26.5 pm/°C.

For the understanding of the radiation effects on the LPG’s temperature sensitivity, theoretical and numerical analysis were carried out in order to estimate the change of the thermo-optic coefficients due to radiation effects. The temperature sensitivity of a LPG can be written as^39^:

2 $$\frac{1}{{\lambda }_{res,i}}\frac{\partial {\lambda }_{res,i}}{\partial T}=\frac{1}{\delta {n}_{eff}}(\frac{\partial {n}_{eff,co}}{\partial T}-\frac{\partial {n}_{cl,i}}{\partial T})+\frac{1}{{\rm{\Lambda }}}\frac{\partial {\rm{\Lambda }}}{\partial T}\cong \frac{1}{\delta {n}_{eff}}(\frac{\partial {n}_{eff,co}}{\partial T}-\frac{\partial {n}_{cl,i}}{\partial T})$$

where *δn*_*eff*_ is the difference between the effective refractive index of the core mode and cladding mode. In most cases, the term related to the silica thermal expansion is several orders of magnitude smaller than the term referred to the thermo-optic effect. This latter term depends on the product of the inverse of the difference between the effective refractive index of core and cladding, and the difference between the thermal sensitivities of the same indices. In our simulations, the effective refractive index of all modes and spectra were computed by considering the dependence of the core and cladding refractive index on the temperature changes to be:

3 $$\{\begin{array}{c}{n}_{co}(\lambda )={n}_{co,0}(\lambda )+{\rm{\Delta }}{n}_{co}^{irr}+({\alpha }_{co}+{\rm{\Delta }}{\alpha }_{co}^{irr}){\rm{\Delta }}T\\ {n}_{cl}(\lambda )={n}_{cl,0}(\lambda )+{\rm{\Delta }}{n}_{cl}^{irr}+({\alpha }_{cl}+{\rm{\Delta }}{\alpha }_{cl}^{irr}){\rm{\Delta }}T\end{array}$$

where *α*_*co*_ and *α*_*cl*_ are the thermal coefficients of the core and cladding respectively (for pure silica^40^ *α* = 7.8∙10^−6^/°C), and *ΔT* is the temperature change.

By comparing numerical and experimental data, we first estimated ${\alpha }_{co}$ and ${\alpha }_{cl}$ for all pre-irradiated gratings, and we found values in perfect agreement with literature^40^. Similarly, for all the gratings, the thermal behaviour after the irradiation can be extrapolated by considering an increase in the thermo-optic coefficient, ${\rm{\Delta }}{\alpha }_{co}^{irr}$ or ${\rm{\Delta }}{\alpha }_{cl}^{irr}$, in the fiber region (core or cladding) exhibiting a non-trivial RI change due to radiation, i.e. non-trivial ${\rm{\Delta }}{n}_{co}^{irr}$ or ${\rm{\Delta }}{n}_{cl}^{irr}$, according to Table 1.

In a similar way to the refractive index change estimation reported in previous sections, we also focused on the experimental variation in thermal sensitivity and we applied the reverse-engineering procedure in order to estimate the corresponding thermo-optic coefficient change. In particular, we found changes in the core thermo-optic coefficient^41^, ${\alpha }_{co}$, lower than 10^−8^/°C for OZ, Thorlabs, and FiberA fibers. Whereas for the Nufern fiber, the same change is about 3∙10^−8^/°C. Finally, for the Draka fiber the change is in cladding thermo-optic coefficient, ${\alpha }_{cl}$, with value of 6∙10^−8^/°C, considering the presence of Fluorine dopant. Table 2 summarizes the changes in LPGs’ thermal sensitivities and thermo-optic coefficients for all the gratings.

**Table 2:** Summary of temperature sensitivity and thermo-optic coefficient changes on LPGs after irradiation.

Sensor id	Experimental data		From numerical-experimental comparison	
	S_T_ [pm/°C] (before irr.)	S_T_ [pm/°C] (after irr.)	${\boldsymbol{\Delta }}{{\boldsymbol{\alpha }}}_{{\boldsymbol{c}}{\boldsymbol{o}}}^{{\boldsymbol{i}}{\boldsymbol{r}}{\boldsymbol{r}}}$	${\boldsymbol{\Delta }}{{\boldsymbol{\alpha }}}_{{\boldsymbol{c}}{\boldsymbol{l}}}^{{\boldsymbol{i}}{\boldsymbol{r}}{\boldsymbol{r}}}$
OZ1-LPG	50.0	51.2	<10^−8^/°C	—
OZ2-LPG	49.9	N.A.	N.A.	N.A.
Thorlabs-LPG	47.6	48.6	<10^−8^/°C	—
FiberA-LPG	50.8	51.6	<10^−8^/°C	—
Nufern-LPG	49.5	57.7	3∙10^−8^/°C	—
Draka-LPG	29.6	26.5	—	6∙10^−8^/°C

## Conclusions

In this work, we experimentally and theoretically investigated the effect of mixed gamma-neutron radiation field on LPGs written by EAD technique. It is the first time, to the best of our knowledge, that LPGs were tested under mixed neutron-gamma radiation in a nuclear reactor. Moreover, these LPGs were fabricated in various optical fibers (standard, specialty, and rad-hard), with different core/cladding composition, in order to compare and better understand their behaviour in nuclear environments. It is important to underline that LPGs were fabricated by means of Electric Arc Discharge technique because it enables the writing of gratings in standard and not photosensitive. Measurements were performed by on-line wavelength shift monitoring during the irradiations and by laboratory spectral characterization. The corresponding changes in optical fiber refractive index and thermo-optic coefficients are also reported. In order to do so, the approach was to combine the experimental measured results with simulations, as obtained from a numerical model accounting both for optical fiber’s and LPG’s parameters, finding good agreement between the two methods.

Previously published reports on the radiation sensitivity of Fiber Bragg Gratings in mixed gamma neutron fields proved they are good candidates for long term (4–8 years) temperature measurements in nuclear reactors^3^, as their radiation sensitivity is much lower than in the case of LPGs, with the Bragg wavelength shift (BWS) lower than 100 pm for neutron fluencies exceeding 10^17^ n/cm^2^. Conversely, it was also found that for specific FBGs’ design like “chemical composition gratings” the response was over 10 nm for thermal/fast neutron fluencies above 7.5·10^19^/10^18^ n/cm^2^ and a gamma-dose higher than 1.5 kGy^42^. Long Period Gratings were not yet tested in mixed gamma neutron field, so no reference data are available. For this reason, the present study is important as it reveals, for all LPGs tested (except of course for the Draka-LPG), a much higher sensitivity to radiation than the one reported for FBGs until now.

Some of the arc induced LPGs reported in the present work have been previously subjected to only gamma irradiation with a ^60^Co source up to saturation levels^22^. In particular, the saturation occurred at the total dose of 35 kGy, with 0.18 kGy/h dose rate, where wavelength shifts of 6.7 nm, 3.7 nm, and 3.9 nm were measured for Nufern-LPG, OZ-LPG, and Thorlabs-LPG, respectively. Whereas, as reported in the current paper, it was found that under mixed gamma-neutron irradiation the corresponding wavelength variations were 11.7 nm, 6.4 nm, and 10.9 nm for the same types of fibers. Tests carried out emphases that the arc-induced LPGs considered herein show a higher radiation sensitivity as compared to LPGs produced by the CO_2_ laser method, as these last samples showed saturation after ^60^Co gamma irradiation from 1.45 nm up to 3.5 nm, at a total dose of 34 kGy^21^.

It should be noticed that very different behaviour was observed between two LPGs in standard fibers provided from different suppliers. This means that the tolerance in the fiber fabrication process can significantly affect the fiber resistance to radiation. Similarly, we found noticeable higher changes in the refractive index of specialty fiber designed to be radiation resistant, such as Nufern. According to literature, we suppose that the radiation resistance characteristics of this fiber are primarily attributed to a low RIA. On the contrary, LPG in pure-silica core Draka fiber exhibits trivial wavelength shifts with respect to the radiation (this fiber has even lower RIA). In particular, by observing the wavelength shift of the LP_04_ cladding mode, values of 6.4 nm, 10.91 nm, 8.99 nm, 11.75 nm, and −0.39 nm are measured for OZ-LPG, Thorlabs-LPG, FiberA-LPG, Nufern-LPG and Draka-LPG, respectively, as consequence of about two hour irradiation with a gamma-ray dose-rate of 9 Gy/s and a mean 1.25∙10^12^ n/(cm^2^s) neutron flux, for a total dose of 64.8 kGy and a neutron fluence of 9.18∙10^15^ n/cm^2^. Referring to the changes in temperature sensitivity, a minor increase of about 1 pm/°C was recorded for standard OZ-LPG, Thorlabs-LPG and specialty FiberA-LPG. In the case of Nufern-LPG temperature sensitivity change was more pronounced, around 8 pm/°C. Finally, concerning Draka-LPG, a decrease in temperature sensitivity of 3 pm/°C was observed.

In conclusion, the higher shifts recorded by Nufern-LPG provides the basis of using them as radiation sensors. Concerning Draka-LPG, the pure-silica core with Fluorine-doped cladding fiber in which the grating was inscribed makes this sensor extremely tolerant to radiation. Moreover, this LPG responded linear with temperature change over a wide range, recommending it as a sound candidate for temperature measurements under extreme radiation fields.
